# A Hospital-Based Work Support Intervention to Enhance the Return to Work of Cancer Patients: A Process Evaluation

**DOI:** 10.1007/s10926-012-9372-2

**Published:** 2012-06-15

**Authors:** S. J. Tamminga, A. G. E. M. de Boer, M. M. E. M. Bos, G. Fons, J. J. E. M. Kitzen, P. W. Plaisier, J. H. A. M. Verbeek, M. H. W. Frings-Dresen

**Affiliations:** 1 Coronel Institute of Occupational Health, Academic Medical Center, University of Amsterdam, PO Box 22700, 1100 DE Amsterdam, The Netherlands; 2 Department of Internal Medicine, Reinier de Graaf Groep, Delft, The Netherlands; 3 Department of Gynaecology, Academic Medical Center, University of Amsterdam, Amsterdam, The Netherlands; 4 Department of Internal Medicine, Albert Schweitzer Hospital, Dordrecht, The Netherlands; 5 Department of Surgery, Albert Schweitzer Hospital, Dordrecht, The Netherlands; 6 Finnish Institute of Occupational Health, Kuopio, Finland

**Keywords:** Cancer patients, Cancer survivors, Return to work, Intervention, Randomised controlled trial, Process evaluation

## Abstract

*Purpose* To perform a process evaluation of a hospital-based work support intervention for cancer patients aimed at enhancing return to work and quality of life. The intervention involves the delivery of patient education and support at the hospital and involves the improvement of the communication between the treating physician and the occupational physician. In addition, the research team asked patient’s occupational physician to organise a meeting with the patient and the supervisor to make a concrete gradual return-to-work plan. *Methods* Eligible were cancer patients treated with curative intent and who have paid work. Data were collected from patients assigned to the intervention group (N = 65) and from nurses who delivered the patient education and support at the hospital (N = 4) by means of questionnaires, nurses’ reports, and checklists. Data were quantitatively and qualitatively analysed. *Results* A total of 47 % of all eligible patients participated. Nurses delivered the patient education and support in 85 % of the cases according to the protocol. In 100 % of the cases at least one letter was sent to the occupational physician. In 10 % of the cases the meeting with the patient, the occupational physician and the supervisor took place. Patients found the intervention in general very useful and nurses found the intervention feasible to deliver. *Conclusions* We found that a hospital- based work support intervention was easily accepted in usual psycho-oncological care but that it proved difficult to involve the occupational physician. Patients were highly satisfied and nurses found the intervention feasible.

## Introduction

Due to the increased survival rates of cancer, a growing number of cancer patients are now be able to survive many years beyond a cancer diagnosis and thus face new challenges related to survivorship. For cancer patients of working age, one challenge is their return to work. Returning to work is important as work contributes to personal [1] and economic well-being, [2] and is associated with the quality of life of cancer patients [3, 4].

Unfortunately, not all cancer patients are able to return to work successfully. A meta-analysis demonstrated that the risk of unemployment was 37 % higher for cancer patients than healthy controls [5]. Moreover, interventions primarily aimed at improving cancer patients’ return to work are rare, especially those that have been studied in randomised controlled trials [6, 7]. Therefore, we developed an intervention on the return to work of cancer patients and quality of life [8].

We developed this intervention based on previous studies that had employed effective interventions for enhancing the return to work of cancer patients [6], and we developed this intervention in collaboration with various stakeholders involved in the return-to-work process of cancer patients [8]. An early intervention is most appropriate, as longer periods of sick leave often cause patients return to work to be more difficult [9, 10]. For the delivery of an early intervention, a hospital-based intervention is most appropriate, as most cancer patients do not have contact with their supervisor or occupational physician during the early phases of their cancer treatment and physician’s advice seems to be influential [11, 12]. The hospital-based work support intervention was developed consistent with the Dutch social security system and carried out in the Netherlands. As return to work is influenced by the institutional context of a country, it is important to understand this context. In short, in the Netherlands, a sick-listed employee receives at least 70 % of their wage, which is paid by the employer. Both the employer and the employee have responsibilities for the return-to-work process. The sick-listed employee cannot be fired due to his/her illness during the first 2 years of sickness absence (Improved Gatekeeper Act).

Performing a process evaluation is important for interpreting the findings of an innovative intervention because the effectiveness partially depends on how well the intervention was implemented [13]. Consequently, process evaluation results can be used to further develop the intervention by improving the intervention itself and/or the intervention implementation.

Process indicators should be measured at each level that could have an influence on the implementation process of the intervention [14]. For instance, intervention exposure occurs in this study on two levels: at the level of cancer patients who received the intervention, and that of nurses who received training for delivering the intervention.

Linnan and Steckler [14] proposed the following key process indicators for studying the intervention implementation: recruitment, context, reach, intensity of the intervention delivered, intensity of the intervention received, and fidelity. In this study, we distinguish between the process indicators that address how well the intervention was delivered and received (intensity of the intervention delivered, intensity of the intervention received (exposure), and fidelity) and those that address how the intervention was appreciated by the various stakeholders (intensity of the intervention received satisfaction), to whom the findings apply (recruitment, reach), and under what conditions the findings can be applied (context). We made these distinctions because the primary aim of the trial was to identify effectiveness of the intervention. The process indicators that address how well the intervention was delivered and received can help us to interpret our findings related to effectiveness, and we therefore considered these the most important process indicators. The remaining process indicators could be helpful when implementing the intervention for usual care on a wider scale. In summary, the objective of this study was to perform a process evaluation of a hospital-based work support intervention for cancer patients.

## Methods

This process evaluation was part of a multi-centre randomised controlled trial to assess the effectiveness of a hospital-based work support intervention on the return to work and quality of life of cancer patients [8]. Patients who were willing and eligible to participate were randomised to either the intervention group and received the hospital-based work support intervention or to the control group and received care as usual [8].

Six hospitals in the Netherlands participated in the study. The medical ethics committee of the Academic Medical Center approved of the study. The local medical ethics committee of each participating hospital advised positively about feasibility of the study in their hospital.

### Patients

Patients with a primary diagnosis of cancer, who were between 18 and 60 years of age, had paid work at the time of diagnosis, were on sick leave, had been treated with curative intent, and who had been treated at one of the participating hospital departments were eligible to participate. Treatment with curative intent was defined as an expected 1-year survival rate of approximately 80 %. We excluded patients, who were not adequately able to speak read, or write Dutch, who had a severe mental disorder or other severe co-morbidity, or those for whom the primary cancer diagnosis had been made more than 2 months ago. Patients signed informed consent forms prior to their inclusion in the study.

### Hospital-Based Work Support Intervention

The hospital-based work support intervention began a few weeks after patients were included in the study and was spread over a maximum of 14 months. The hospital-based intervention involves delivery of patient education and support at the hospital integrated into usual psycho-oncology care and involves improvement of the communication between the treating physician and the occupational physician. In addition, the research team asked patient’s occupational physician to organise a meeting with the patient and the supervisor to make a concrete gradual return-to-work plan [8]. A nurse who delivered psycho-oncological care in normal cancer care delivered patient education and support at the hospital in 4 meetings of 15 min each. Nurses received a half-day training course in which the intervention protocol was simulated. In addition, three letters were sent to the occupational physician to enhance the communication: two from the treating physician and one from the nurse. The key aspects of the hospital-based work support intervention were the patient education and support at the hospital and the sending of information to the occupational physician. In the Netherlands, patients must give their consent to allow medical information to be sent from a treating physician to an occupational physician, which was requested by the nurse during the first meeting. The research team only informed occupational physicians about diagnosis and cancer treatment of patients who gave consent providing medical information to their occupational physician.

### Process Evaluation

**Fig. 1:**
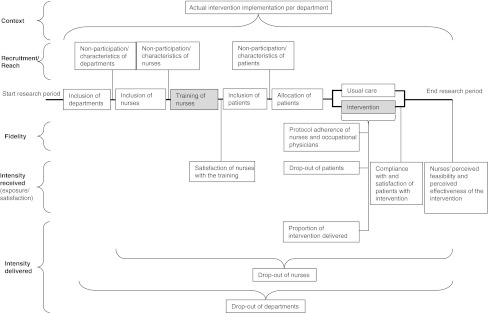
Process indicators. *Legend*: *Grey* denotes intervention delivery

In accordance with the key process indicators that had been proposed by Linnan and Steckler [14], we measured the following aspects: recruitment, context, reach, intensity of the intervention delivered, intensity of the intervention received, and fidelity. The various time points for the data collection of data regarding the process indicators are shown in Fig. 1.

### Study Design

Data of the process indicators were collected using questionnaires, which were filled in by nurses and patients, nurses’ reports of each patient in the intervention group, and checklists that were filled in by the research team throughout the study (Table 1).

**Table 1:** Process indicators

Process indicators Definition	Measurement level	Measurement	Measurement tool
*Recruitment* The proportion of hospitals, hospital departments, and nurses who participated in the study compared to the number of the hospitals, hospital departments, and nurses that were contacted by the research team	Hospital department	Participation (yes or no) and reason for non-participation	Checklist
	Nurse	Participation (yes or no) and reason for non-participation	
*Context* The contextual aspects (e.g. usual cancer care) that directly or indirectly affect the intervention implementation	Hospital department	Cancer diagnosis Occupational of health care professional who delivered patient education and support at the hospital	Checklist
*Reach* The extent to which the target population participated in the intervention	Cancer patients	Proportion of cancer patients that did participate compared to all eligible cancer patients	Checklist
*Intensity of the intervention delivered* The extent to which the intervention actually was delivered according to the intervention protocol	Hospital department	Drop-out rate of hospital departments	Checklist
	Hospital department	Proportion of intervention that was delivered according to the intervention protocol based on number of meetings, number of meetings face-to-face, and duration of each meeting	Nurses reports
	Nurse	Drop-out rate of nurses	Checklist
*Intensity of the intervention received* The extent to which the intervention was actually received by the target population			
Exposure	Patient	The number of advices that a patients complied with^a^	Questionnaire^b^
	Patient	Drop-out rate of patients	Nurses report
Satisfaction	Nurse	Satisfaction with the training for delivering the intervention	Questionnaire^c^
	Nurse	Perceived feasibility of the intervention	Questionnaire^c^
	Nurse	Perceived effectiveness of the intervention	Questionnaire^c^
	Patient	Satisfaction with the intervention^a^	Questionnaire^b^
*Fidelity* The extent to which the intervention content was carried out according to the intervention protocol			
Protocol adherence Based on 6 performance indicators	Nurse	1. Whether the quality of the meetings between the nurse and the patient was adequate 2. Whether the nurse delivered sufficient information to the patient 3. and 4. Whether medical information was sent to the patient’s occupational physician	Nurses reports
	Occupational physician	5. Whether the occupational physician organised a meeting with the patient, patient’s supervisor, and him/herself 6. Whether a return-to-work plan was made in collaboration with the patient, patient’s supervisor, and the occupational physician	

^a^Measured 14 months after randomisation

^b^Consisted both of closed and open-ended questions

^c^Consisted of open-ended questions only

### Measurement Level

Process indicators were measured at three levels (Table 1) and these included the hospital department in which the intervention was carried out, nurses and occupational physicians who delivered the intervention, and patients assigned to the intervention group. Only patients assigned to the intervention group were included in this process evaluation, as patients assigned to the control group received care as usual only. Patients were asked to fill in a questionnaire 14 months after randomisation, which were sent to patients’ home with a free return envelope enclosed. Nurses reported on each patient assigned to the intervention group after each meeting. Nurses that delivered the intervention to at least five patients were asked to fill in a questionnaire after the study was completed, which were sent to nurses’ work with a free return envelope enclosed (N = 4).

The response rate of patients to the questionnaire was 75 % (N = 49). Reasons for not responding included cancer recurrence (N = 2), study decline (N = 3), or were unknown (N = 11) and two patients died before the intervention was completed. Nurses’ reports for 6 (10 %) patients who received at least one nurse consultation were lost and nurses’ response rate to the questionnaire was 100 %. The research team collected reach data from three hospital departments (A, C, and E) only. The other hospital departments were not able to provide data on reach due to time constraints.

**Fig. 2:**
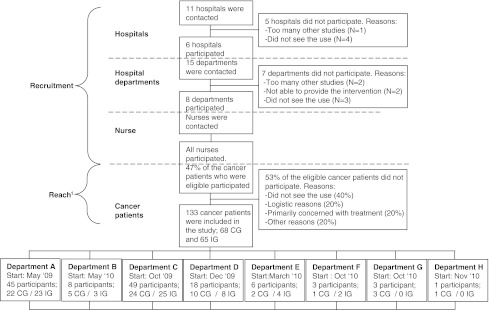
Recruitment and reach. *CG* control group, *IG* intervention group. 1 Based on three departments (A, C, E)

Of the 8 hospital departments that participated in the study, 2 hospital departments (G and H) did not treat patients who were assigned to the intervention group (Fig. 2). Therefore, the process indicators context, intensity of the intervention received, intensity of the intervention delivered, and fidelity were only assessed in 6 hospital departments (A–F).

### Process Indicators

#### Recruitment

We measured participation (yes or no) of hospitals, hospital departments, and nurses, as well as reasons for non-participation. We measured recruitment as the proportion of hospitals, hospital departments, and nurses that did participate in the study compared to the total number of hospitals, hospital departments, and nurses that had been contacted by the research team.

#### Context

We measured intervention implementation per department to identify whether various health care contexts directly or indirectly affected intervention implementation. Two factors were considered important during the research period: type of cancer diagnosis and occupation of the health care professional who delivered the patient education and support at the hospital.

#### Reach

Reach was measured to identify to what extent the target population participated in the study and to identify if our procedure to reach patients was feasible. Reach was expressed as the proportion of cancer patients that did participate in the study compared to all cancer patients that were found eligible to participate. Furthermore, we registered age of the cancer patients who did and did not want to participate in order to identify if our findings apply to all age groups.

#### Intensity of the Intervention Delivered

We measured the proportion of the intervention that was actually delivered compared to the intervention protocol as intensity of the intervention delivered for patients who started with the intervention. For each patient in the intervention group, intensity of the intervention delivered was measured as the number of meetings that were held, the number of meetings face-to-face, and their duration. We protocolised the delivery of 4 meetings of at least 10 min each of which at least two meetings were face-to-face unless a patient reached sustainable return to work before the intervention was completed. At least one initial meeting for all patients was protocolised regardless of work resumption. In case a patient was unable to complete the intervention due to medical reasons (i.e. cancer recurrence), we considered the intervention as delivered according to the protocol. In addition, drop-out of hospital departments and nurses was recorded.

#### Intensity of the Intervention Received (Exposure)

We measured exposure to the intervention as the extent to which the intervention was received as intended by measuring whether patients complied with the advice that was provided (yes or no). For each type of advice offered, an open-ended question was directed to patients, whereby patients could provide their source of motivation for not complying with the advice. We considered compliance with 50 % of the provided advice as sufficient. In addition, patient drop-out (yes or no) and their reason for dropping out, as well as the characteristics of the patients (e.g. educational level and income) were recorded in order to identify whether compliance applied to the entire population.

#### Intensity of the Intervention Received (Satisfaction)

Satisfaction with the intervention was assessed at both nurse and patient level.

*Nurses’ satisfaction with the training:* Nurses’ satisfaction regarding the training they had received for delivering the intervention was measured and all questions (N = 5) were open-ended questions.

*Nurses’ perceived feasibility of the intervention:* Nurses were asked whether they thought that the intervention was applicable in practice and whether they encountered barriers when applying the intervention in practice and how to best overcome these barriers in the future. Finally, we identified the nurse satisfaction with the intervention protocol and all of these questions (N = 4) were open-ended questions.

*Nurses’ perceived effectiveness of the intervention:* Nurses were asked whether they thought the intervention was effective at enhancing the return to work of cancer patients. They were also asked which portion of the intervention they considered most useful and which not, and for which population of cancer patients. All questions (N = 14) were open-ended questions.

*Patients’ satisfaction with the intervention:* Patient’s were asked about their satisfaction with each intervention component, the timing of each intervention component, the duration of the intervention, and the competence of the nurse and the occupational physician. Furthermore, if the intervention fulfilled their expectations, the perceived burden, and whether the timing of the intervention was adequate were also assessed, using 3- and 4-point Likert scales as well as open-ended questions.

#### Fidelity

Fidelity refers to the extent to which the intervention content was carried out according to the protocol. We measured fidelity by assessing performance of nurses and occupational physicians based on the intervention protocol, i.e. protocol adherence. Six performance indicators were established a priori based on the intervention protocol. An independent researcher assessed protocol adherence by scoring each indicator as either sufficient or insufficient, or not applicable. All performance indicators were weighted equally, yielding a maximal sum score of 6.

The first two performance indicators assessed nurses’ performance and were assessed based on the reports that the nurses completed after each meeting with a patient. The first performance indicator addressed whether the quality of meetings between the nurse and the patient was adequate. The second performance indicator addressed whether nurses delivered sufficient information to a patient. The third and fourth performance indicators assessed whether medical information was sent to the patient’s occupational physician (yes or no) and a score was assigned if this had taken place. The fifth and sixth performance indicators assessed occupational physicians’ performance on the basis of nurses’ reports. The fifth performance indicator assessed whether the occupational physician organised a meeting with the patient, patient’s supervisor, and him/herself, and the sixth performance indicator assessed whether a return-to-work plan was draw-up in collaboration with the patient, patient’s supervisor, and the occupational physician.

### Statistical Analysis

All quantitative data were analysed with descriptive statics using PASW version 18 [15]. Differences between patients who participated and those who did not, regarding age were analysed with Student’s t test. Differences regarding educational level and income between patients who demonstrated at least 50 % compliance with the provided advices and those who demonstrated compliance below 50 % were analysed with Mann–Whitney U test for educational level and Student’s t test for income. A p value of ≤0.5 was considered statistically significant.

The open-ended questions were qualitatively analysed by the first author using content analysis [ST] and were checked by another independent researcher. Codes were derived from the open-ended questions and were categorised. Codes represent the text as closely as possible.

## Results

The recruitment of hospitals and hospital departments initially occurred between September 2008 and December 2009 but the recruitment period was extended by 4 months to include as many cancer patients as possible. The onset of the study per department occurred between May 2009 (department A) and November 2010 (department H) and ended at the end of December 2010 for all hospital departments (Fig. 2).

Of the 133 patients included in the study, 65 patients were assigned to the intervention group. The baseline characteristics of these 65 patients are presented in Table 2. Patients were on average 47.5 ± 8.2 years old, and all patients but one were female. Sixty-four percent of the patients were diagnosed with breast carcinoma, 31 % were diagnosed with gynaecological forms of cancer and 5 % of the patients were diagnosed with other forms of cancer.

**Table 2:** Baseline characteristics of patients assigned to the intervention group

Patient characteristic^a^	Intervention group (N = 65)
*Socio-demographic characteristics*	
Age (years)^b^	47.5 ± 8.2
Gender (% female)	99 %
Marital status (% married or living with partner)	79 %
Breadwinner position (% sole or shared)	65 %
Education level (%)	
Low	11 %
Intermediate	59 %
High	30 %
*Clinical characteristics*	
Diagnosis (%)	
Mamma carcinoma	64 %
Cervix carcinoma	23 %
Ovarian carcinoma	5 %
Vulva carcinoma	3 %
Other	5 %
Days since diagnosis	48.1 ± 35.6
*Work-related characteristics*	
Type of occupation (%)	
Public health	38 %
Administrative	9 %
Sales	5 %
Other	48 %
Type of work (% mainly physically demanding work)	32 %
Time since sick listed (days)	26.5 ± 35.1
Number of working hours according to contract (1–40)	26.4 ± 8.9
Importance of work (VAS) (0–100)^c^	58.7 ± 23.1
Shift work (% shift work)	26 %
*Type of contract (%)*	
Permanent	89 %
Temporary	11 %
Overall work ability (WAI) (0–10)^c^	5.3 ± 3.0
Work ability physical work load (WAI) (0–5)^c^	3.5 ± 1.1
Work ability mental work load (WAI) (0–5)^c^	3.0 ± 1.06
*Health-related characteristics*	
Quality of life (VAS) (0–100)^c^	59.7 ± 21.7
General fatigue (MFI) (0–20)^c^	12.4 ± 4.9
Depression (CES-D) (0–60)^c^	14.1 ± 9.3
Self-efficacy (ALCOS) (0–80)^c^	66.5 ± 8.6

^a^Continuous variables: mean ± SD; nominal and ordinal variables percentages

^b^Age at the time of randomisation

^c^Higher score means a higher level of importance of work, work ability, quality of life, fatigue, feelings of depression, and self-efficacy

Of the 65 patients assigned to the intervention group, 58 (89 %) patients received at least one consultation with the nurse to receive patient education and support about return to work, of 54 (100 %) patients at least one letter was sent to their occupational physician, and the meeting between the supervisor and the occupational physician to draw-up a return-to-work plan occurred in 5 (10 %) cases. Reasons for not receiving the patient education delivered by the nurse included logistical issues related to their treatment in another hospital department (N = 6) or a lack of interest (N = 1) (Fig. 2).

### Recruitment

Of the 11 hospitals that were contacted by the research team, 5 hospitals did not participate in the study (Fig. 2). Reasons for non-participation included, uncertainty about the benefits of providing patient education and support regarding return to work as part of psycho-oncological care (N = 3), a large number of other studies conducted (N = 1), and a reluctance to asks cancer patients to participate in a study about return to work soon after their cancer diagnosis (N = 1). There were 6 hospitals that decided to participate, and 7 of the 15 hospital departments that were contacted by the research team decided not to participate. Reasons for hospital department non-participation included the existence of a large number of other ongoing studies (N = 2), nurses being unable to deliver patient education and support about return to work due to time constraints or limited psycho-oncological care (N = 2), an inability to include cancer patients prior to their initial cancer treatment (N = 2), and the uncertainty about the benefits of providing patient education and support about return to work as part of psycho-oncological care (N = 1). In sum, 8 departments from 6 hospitals participated in the study.

At the onset of the study, 6 of the 8 hospital departments employed only one person who could deliver psycho-oncological care as well as the intervention, although each of these individuals were willing to deliver the intervention. In hospital departments where more than one person delivered psycho-oncological care, the supervisor of each department decided which persons would be able to deliver the intervention based on their years of experience. All nurses, who were eligible to deliver the intervention, were willing to participate.

### Context

Five hospital departments (83 %) treated breast cancer patients and one department (17 %) treated gynaecological cancer patients. Breast-care nurses delivered the intervention in three hospital departments (50 %), an oncology nurse in one department (17 %), a nurse practitioner in one department (17 %), and a medical social worker in another (17 %).

### Reach

Based on the findings from three hospital departments (A, C, and E), an average of 47 % of the eligible cancer patients participated in the study (Fig. 2). Reasons for cancer patients not to participate included, not seeing a use of the intervention (40 %), logistical reasons (20 %), having other things on their mind (20 %), or other reasons (20 %). Age of the patients who did and did not participate did not differ statistically (*p* = 0.2).

### Intensity of the Intervention Delivered

None of the 6 hospital departments dropped out of the study, although one of the nurses dropped out of the study due to a career change. This nurse’s tasks related to delivering the intervention were completed by one of the other nurses, and as such, the intensity of the intervention delivered was not affected. Fifty-seven percent of the patients had 4 meetings, 66 % three meetings, 76 % two meetings, and 88 % had one meeting (Table 3). In addition to these meetings, 15 % of the patients had an additional meeting with their nurse to receive extra support for their return to work.

**Table 3:** Intensity of the intervention delivered—proportion of the intervention that was delivered

Department Patients assigned to the intervention group of which we had nurses report	A–F (N = 59)	According to the intervention protocol (% according to the protocol)
*Intensity of the intervention delivered*		
Number of meetings N (%)		
4 Meetings	34 (57 %)	88 %
3 Meetings	39 (66 %)	
2 Meetings	39 (76 %)	
1 Meeting	52 (88 %)	
Type of contact N (%) meetings face-to-face		
Meeting 1	35 (81 %)	63 %
Meeting 2	22 (63 %)	
Meeting 3	12 (38 %)	
Meeting 4	5 (19 %)	
Duration of meetings in minutes Median (range)		
Meeting 1	20 (10–60)	97 %
Meeting 2	20 (9–60)	
Meeting 3	25 (7–45)	
Meeting 4	18 (10–60)	

Eighty-one percent of the patients had the first meeting face-to-face, 63 % had the second meeting face-to-face, 38 % had the third meeting face-to-face, and 19 % had the fourth meeting face-to-face (Table 3). Duration of meetings between the nurse and the patient was on average 21 min and ranged between 7 and 60 min (Table 3). For 88 % of the patients, meetings were delivered in accordance with the intervention protocol. For 63 % of the patients, face-to-face meetings were delivered in accordance with the intervention protocol, and duration of the meetings was in accordance with the study protocol for 97 % of the patients.

### Intensity of the Intervention Received (Exposure)

Patient compliance with the advice to keep in contact with employer (79 %), to keep in contact with co-workers (79 %), and the advice to start with return to work before full recovery (75 %) were complied with the most (Table 4). The advice to evaluate the return-to-work plan with their supervisor (52 %) and the advice to draw up a second return-to-work plan were complied with the least (33 %).

**Table 4:** Intensity of the intervention received (exposure)

Department Patients assigned to the intervention group who filled in questionnaire	A–F (N = 24)
*Intensity of the intervention received*	
Percentage advices acted upon *N (* *%)*	
Make appointment with OP	12 (63 %)
Keep in contact with employer	19 (79 %)
Keep in contact with co-workers	19 (79 %)
Draw up return-to-work plan with supervisor and OP	16 (70 %)
Start to return to work before full recovery but with limited number of hours	18 (75 %)
Make sure that the return-to-work plan encompasses the data and number of hours of start, which days of the week will be worked, the timing of the expansion of hours, the tasks and number of hours of this expansion, and the proposed date of full return to work	14 (58 %)
Evaluate return-to-work plan with supervisor every 2 weeks	12 (52 %)
Draw up a second return-to-work plan that may be used if the first plan fails	8 (33 %)

*OP* occupational physician

From the open-ended questions of the patients we inferred that non-compliance with the advice to schedule a meeting with the occupational physician, to keep in contact with employer, and to keep in contact with co-workers was caused by either the fact that it was common practice (N = 10) or because a patient did not have an employer anymore (N = 1). In addition, the open-ended questions revealed that patients’ did not comply with the advice to make a return-to-work plan for various reasons, including did not have an employer anymore (N = 1), already made a return-to-work plan (N = 1), or still have to make a return-to-work plan (N = 1). Not complying with the advice to draw up a second return-to-work plan was caused by not seeing the use of doing it (N = 3).

The education and income level of patients who demonstrated at least 50 % compliance versus those who demonstrated less than 50 % compliance did not differ statistically (*p* = 0.3–0.8). All but one nurse received the training for how to deliver the intervention and this nurse did not receive the training due to a time constraint.

### Intensity of the Intervention Received (Satisfaction)

Nurses scored the training they received with a mean score of 8 on a scale from 0 (very poor) to 10 (very good). The open-ended question responses indicated that some nurses (N = 3) would have preferred to receive the training material before the start of the training and that some nurses thought the period between the training and the start of the intervention was too long (N = 2).

All nurses (N = 4) were satisfied with the intervention protocol and stated that it provided a clear overview of the content of the intervention. In general, nurses (N = 4) believed that the intervention was feasible to carry out in practice and that the burden associated with the delivery of the intervention was manageable. Nevertheless, the following barriers for applying the intervention to practice were mentioned: (1) delivering the intervention for patients who did not receive usual psycho-oncological care; (2) delivering the intervention by telephone; and (3) integrating the intervention into usual care. For the first barrier, nurses mentioned (N = 3) that the intervention was not as feasible to deliver to patients who did not receive usual psycho-oncological care. This situation may have occurred for patients, who did not receive follow-up care at the hospital, but for the delivery of the intervention in these cases, an extra consultation was planned or meetings were held by telephone.

Second, delivering the intervention by telephone was perceived as less feasible because it was time consuming to reach patients by telephone and it was difficult to assess the patient’s situation and gain patient’s trust over the telephone. Third, nurses (N = 4) stated that the intervention should have been integrated into usual care according to the following adaptations: (1) meetings needed to be planned at the right time and for the proper length of time; (2) all meetings should have been face-to-face; and (3) to be able to deliver all meetings face-to-face it may mean that the intervention should be handed on to another health care professional who would be able to conduct longer follow-up consultations.

Although all nurses (N = 4) believed that most patients benefited from the intervention, some nurses expected (N = 2) the intervention to be only moderately effective because they felt that their advice and support may not have uniquely impacted the return to work of cancer patients, as these patients typically arrange their return to work at the workplace with their supervisor and occupational physician. However, nurses (N = 4) did consider the intervention to be useful for all cancer patients of working age.

Patient satisfaction regarding the various intervention components and their timing is shown in Table 5. Of all patients, 78 % found the timing of their inclusion in the study appropriate, 80 % described the duration of the intervention as adequate, and 98 % of the patients found the burden related to intervention participation small or acceptable. The content of meetings with the nurses were on average perceived by 95 % of the patients as useful or somewhat useful (range 88–100 %). Furthermore, on average, 84 % of the patients perceived the informational leaflet and the 10-steps of advice as useful or somewhat useful (range 63–100 %). The meeting with the supervisor and the occupational physician was perceived by 88 % of the patients as useful or somewhat useful. Furthermore, an average of 70 % of the patients perceived the timing of the various intervention components to be appropriate (range 63–73 %), whereas the remaining patients indicated that they would have preferred these components to be delivered later.

**Table 5:** Intensity of the intervention received (satisfaction)

Department Patients assigned to the intervention group who filled in questionnaire and who reported receiving intervention component	A–F (N = 45)
Intervention	
Timing being asked to participate N (%)	
Right time	35 (78 %)
Too soon	9 (20 %)
Too late	1 (2 %)
Duration of the intervention N (%)	
Right time	31 (80 %)
Too short	7 (18 %)
Too long	1 (3 %)
Burden to participate in the intervention N ( %*) small or acceptable*	40 (98 %)
Meetings with nurse N ( %) useful or somewhat useful	
Competence of the nurse N ( %) good or acceptable	39 (93 %)
Appreciated meetings at the hospital N ( %) yes or somewhat	38 (93 %)
Discuss importance of work	36 (95 %)
Discuss working through cancer treatment	31 (97 %)
Discuss method to disclose cancer diagnosis to supervisor/colleagues	23 (92 %)
Discuss return to work	28 (88 %)
Discuss return-to-work plan	18 (100 %)
Discuss work situation at follow-up	15 (100 %)
Information N ( %) useful or somewhat useful	
Information leaflet	37 (100 %)
10-Steps of advice	
Make appointment with OP	15 (63 %)
Keep in contact with employer	18 (75 %)
Keep in contact with co-workers	18 (75 %)
Draw up return-to-work plan with supervisor and OP	22 (92 %)
Start to return to work before full recovery but with limited number of hours	21 (88 %)
Include detailed information in return-to-work plan	22 (96 %)
Provides information on the prognosis of return to work	22 (92 %)
Evaluate return-to-work plan with supervisor every 2 weeks	22 (92 %)
Draw up a second return-to-work plan that may be used if the first plan fails	16 (67 %)
Provides an example of a return-to-work plan	21 (88 %)
Meeting with OP and supervisor	
Competence of the OP N (%) good or acceptable	39 (81 %)
Competence of the supervisor N (%) good or acceptable	39 (83 %)
Useful meeting OP and supervisors N (%) agree or somewhat agree	16 (88 %)
Supervisor collaborated N (%) yes or somewhat	14 (93 %)
OP collaborated N (%) yes or somewhat	12 (86 %)
Agree with return-to-work plan N (%) yes or somewhat	12 (92 %)
Able to carry out return-to-work plan N (%) yes or somewhat	11 (85 %)
Timing of the intervention components	
Information leaflet N (%) right time	25 (71 %)
10-Steps of advice N (%) right time	15 (63 %)
Discus return to work with nurse N (%) right time	22 (71 %)
Meeting OP and supervisor N (%) right time	11 (73 %)

*OP* occupational physician

### Fidelity

The median sum score of the performance indicators that met the a priori formulated criteria was 4 and ranged between 0 and 6. The performance indicator for sending medical information to the occupational physician (100 %), the indicator for satisfactory quality of meetings between the nurse and the patient (88 %), and the indicator for the delivery of sufficient information to the patients (83 %) were met in most cases (Table 6). The performance indicator for the meeting between the patient, supervisor, and occupational physician to draw-up a return-to-work plan had a frequency of 10 %. Reasons for why nurses did not adhere to the protocol included its perceived usefulness or time constraints.

**Table 6:** Fidelity—protocol adherence

Department Fidelity	Performance indicator ( % positive score)	A–F
Patients assigned to the intervention group of which we received nurse’ s report (N = 56)	Satisfactory quality of meetings between nurse and patient	44 (88 %)^a^
	Nurse provided sufficient information to patient	43 (83 %)^a^
Patients assigned to the intervention group who gave consent to send medical information to OP (N = 54)	Nurse sent information to OP	14 (26 %)
	Medical information from treating physician to OP	54 (100 %)
Patients assigned to the intervention group of which we received nurse’s report and who gave consent to send medical information to OP (N = 48)	Meeting between patient, supervisor, and OP	5 (10 %)
	Drawing up return-to-work plan with patient, supervisor, and OP	5 (10 %)

^a^For three patients the performance indicators were not applicable due to cancer recurrence

*OP* occupational physician

## Discussion

The objective of this study was to perform a process evaluation of a hospital-based work support intervention. A total of 47 % of all eligible patients participated (reach) and nurses delivered patient education and support according to the protocol in 85 % of the cases (fidelity). In 100 % of the cases, at least one letter was sent to the occupational physician (fidelity) and in 10 % of the cases, the meeting with the patient, the occupational physician, and the supervisor took place (fidelity). We found that a hospital-based work support intervention was easily accepted into usual psycho-oncological care but that it was difficult to involve the occupational physician. Overall, patients were highly satisfied, and nurses found the intervention to be feasible.

### Strengths and Limitations

The strength of our study was the thorough analysis of the process indicators at the department, nurse, and patient level, which was based on a previously established framework for process evaluations [14].

One limitation of our study was that we did not include occupational physicians in the data collection process. However, the key aspects of the hospital-based work support intervention were both the patient education and support delivered by a nurse at the hospital, and communication with patient’s occupational physician concerning patient’s diagnosis and treatment. Because earlier research had shown that occupational physicians appreciated receiving this type of information from the hospital [16, 17] and because half of the occupational physicians in this previous study indicated that the information had influenced their rehabilitation efforts [16], we thought that assessing these aspects in the current study was not necessary.

Another limitation of our study was the method that was used to measure fidelity. We measured fidelity by scoring performance indicators based on self-reports of nurses and we do not know how valid these self-reports are in comparison to independent observations. Thus, bias could have been introduced by the recording of socially desirable answers in the reports. However, independent observation may have introduced another form of bias as well, as nurses may have performed differently if they knew they were being observed. Another limitation of the study was the potential for recall bias, as the participants’ compliance and satisfaction with the intervention were assessed at the end of the follow-up period. However, we could not have evaluated these aspects directly after the consultation, because this may have influenced the effect of the intervention in cases in which the patient did not receive the information during the consultation but rather received the information as during the response to the questionnaire. Finally, selection bias may also have occurred, as not all patients responded to the questionnaire. We do not know whether reasons for not completing the questionnaire were related to patient satisfaction or compliance with the intervention. Therefore, it is possible, that these results represent either an overestimation or underestimation.

### Comparisons with the Literature

Nieuwenhuijsen et al. [16] studied the feasibility of an intervention for cancer patients consisting of enhanced provider communication and patient education. In comparison to this study, our study demonstrated similar level of patient satisfaction with the 10-steps of advice, whereas we found a bit lower percentage of patient compliance with the advice provided. We assume that this discrepancy was caused because some of theses advices had become common practice.

Almost 50 % of eligible patients participated in our study, which was considered an adequate result because it should be taken into account that participants had been diagnosed with cancer only a few weeks before the start of the study and therefore experienced higher levels of insecurity. Similar response rates were reported for the inclusion of recently diagnosed cancer patients in a life-style intervention trial [18]. For other types of patients and for other types of interventions, higher response rates have been reported. One Dutch study found a higher reach for patients with low back pain in a trial aimed at preventing work disability [19]. However, this response rate was likely overestimated because it was not based on all of the eligible patients who were invited to participate. Based on our value of reach and the opinions of patients, we can infer that work is a relevant topic for cancer patients also already early in the course of their disease. We also assume that under conditions of regular care rather than trial conditions; reach would further improve, as patients in these conditions do not have to decide about all the extras of a trial such as meeting with a researcher for informed consent and filling in questionnaires.

Other comparable trials for work support interventions among cancer and other patients have also reported the results of process evaluations [20–22]. These evaluations measured the adherence of occupational physicians to the intervention protocol. Verbeek et al. [20] reported an adherence rate that varied from 3 % to 78 % regarding the provision of advice to cancer patients about their return to work. Nieuwenhuijsen et al. [22] reported that only 10 % of the patients received optimal care when their absence from work was a result of mental health problems. Rebergen et al. [21] reported that, on average, adherence of physicians was 50 %, with a maximum adherence score of 20. For our intervention, average adherence of nurses was 85 %, and this result was very good in comparison to these studies. Although these previous studies reported on protocol adherence of occupational physicians and we reported on protocol adherence of nurses, both of these groups were the healthcare providers who delivered the work support intervention, and the results are therefore comparable.

### Interpretations of Findings

Our study showed that the various health care contexts (e.g. cancer diagnosis or type of health care professional) did not influence the intervention implementation. This finding indicates that our intervention could be successfully adapted to various health care contexts, provided that some form of psycho-oncological care is available. This means that our intervention might be successfully adapted to other countries, despite the variation between the social security systems. However, the content and timing of the intervention should be adapted to each social security system.

The intensity of the present intervention delivered was high and was also concurrent with what we protocolised. Few drop-outs were noted, and nurses were able to extend their consultation to deliver the intervention. In contrast, the number of patients who did not start with the intervention was higher than anticipated, which was mainly caused by the fact that those patients did not receive usual psycho-oncological care from nurses who delivered the intervention. For these patients, nurses encountered problems and either and extra consultation was required or the intervention had to be delivered completely over the telephone. Nurses considered this form of delivery to be less effective and more difficult. We believe that this situation would be remediated if the intervention could be implemented over a wider scale, which would provide usual psycho-oncological care to all patients and better integrate the intervention with patient care.

Patients and nurses were in general very satisfied with the various intervention components and found that the timing of the intervention components was appropriate. However, encouraging the occupational physicians to organise a meeting between the patient, the supervisor and him/herself in order to draw-up a return-to-work plan proved difficult, which was likely the result of not actively involving the occupational physician into the hospital-based work support intervention.

As expected, patient compliance with each type of advice provided was high. Only patients with a temporary employment contract that could not be extended were unable to comply with the delivered advices, as they no longer had an employer/occupational physician.

### Implications for Further Research and Practice

In terms of clinical practice, this study demonstrated that psycho-oncological care can address the work concerns of cancer patients at an early treatment phase as well as during follow-up, according to the reported satisfaction of patients and nurses who provided the intervention. However, for further improvement, nurses suggested the following adaptations: 1) meetings should be planned at the right time for the proper length of time; 2) meetings should be conducted face-to-face; and 3) to be able to deliver all meetings face-to-face it may mean that the intervention should be hand on to another health care professional who have longer follow-up consultations in usual cancer care.

Our study was restricted to breast and gynaecological cancer patients. However, nurses who delivered the intervention indicated that all cancer patients of working age would likely benefit from this type of intervention. Thus, evaluation studies of patients with other types of cancer are needed.

It proved difficult to involve the occupational physician and the supervisor in the intervention. As these individuals are relevant to return to work of cancer patients [8, 23], further research is required to increase their involvement. Due to the relatively low prevalence of cancer at the workplace and because most contacts during early phases of treatment are with health care professionals at the hospital, we believe that it would be difficult to organise a workplace-based intervention. However, methods to involve the workplace in the intervention should be extended, for example involving the occupational physician and the supervisor may be achieved by the sending of coded emails instead of letters to decrease the barrier to reach each other. However, patient’s privacy should be guaranteed at all times.

Because patients with a temporary employment contract could not comply with the advices provided, the intervention should be adapted for patients with this type of employment by assessing the specific needs and concerns of this population. This approach is especially important, as patients with a temporary employment contract have a higher risk of becoming unemployed in comparison to patients with a permanent employment contract [24, 25] and because the labour market is changing towards a higher frequency of temporary employment contracts [26].
